# A global view of the human post-translational modification landscape

**DOI:** 10.1042/BCJ20220251

**Published:** 2023-08-23

**Authors:** Naoya Kitamura, James J. Galligan

**Affiliations:** Department of Pharmacology and College of Pharmacy, University of Arizona, Tucson, Arizona 85721, U.S.A.

**Keywords:** acetylation/deacetylation, acylation, glycation, mass spectrometry, methylglyoxal, post-translational modification

## Abstract

Post-translational modifications (PTMs) provide a rapid response to stimuli, finely tuning metabolism and gene expression and maintain homeostasis. Advances in mass spectrometry over the past two decades have significantly expanded the list of known PTMs in biology and as instrumentation continues to improve, this list will surely grow. While many PTMs have been studied in detail (e.g. phosphorylation, acetylation), the vast majority lack defined mechanisms for their regulation and impact on cell fate. In this review, we will highlight the field of PTM research as it currently stands, discussing the mechanisms that dictate site specificity, analytical methods for their detection and study, and the chemical tools that can be leveraged to define PTM regulation. In addition, we will highlight the approaches needed to discover and validate novel PTMs. Lastly, this review will provide a starting point for those interested in PTM biology, providing a comprehensive list of PTMs and what is known regarding their regulation and metabolic origins.

## Introduction

**Figure 1.**
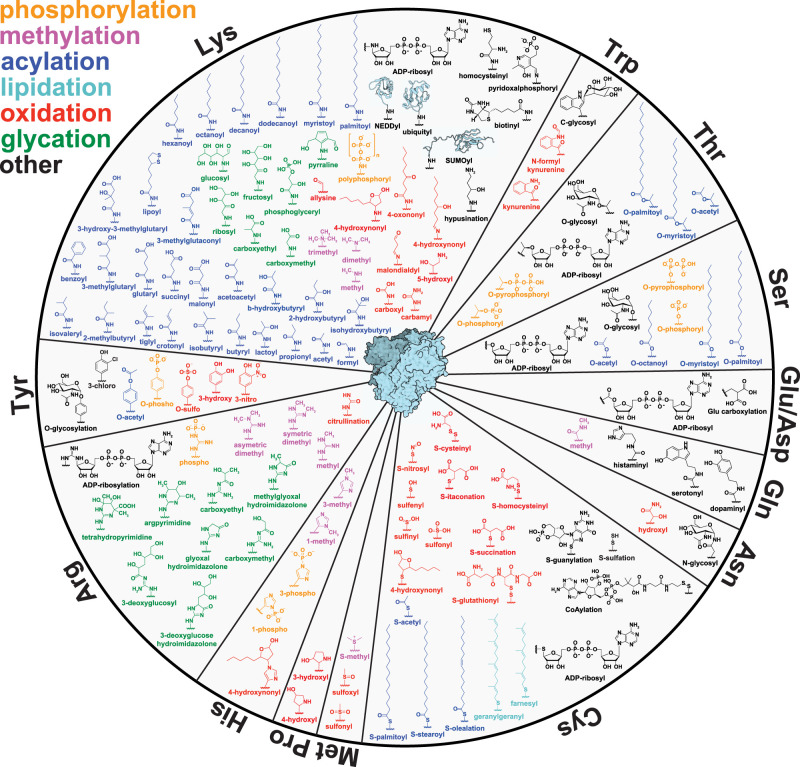
A comprehensive view of the PTM landscape in the human proteome.

The ability of cells to rapidly detect and respond to stimuli is critical to maintaining homeostasis [1,2]. As protein synthesis is an energy demanding and slow process, altering protein function through the addition and removal of small molecules to target proteins provides a rapid response to restore homeostasis. This integration of post-translational modifications (PTMs) significantly expands the functional proteome, which estimations suggest to be >1 000 000 proteoforms from ∼20 000 genes [3]. PTMs are often derived from primary or secondary metabolic intermediates, serving as sensors for cellular nutrient status and often showing conservation across all domains of life [4–6]. The list of known PTMs is expansive, varying in target amino acid, chemical composition, and metabolic source (Figure 1 and Table 1) [6]. While some PTMs are well-characterized (e.g. Ser/Thr phosphorylation), many remain scantly investigated and their role in health and disease is largely unknown (e.g. His methylation).

**Table 1.** PTMs found in the human proteome

**PTM Class**	**Analyte**	**Variable Mass**	**Metabolic Precursor**	**Writer**	**Eraser**	**Reference**
**Methylation**	methyl	Arg + 14.0157	S-adenosylmethionine	Type I, II, III PRMTs	KDM4E, KDM5C	[148-153]
		Gln + 14.0157	S-adenosylmethionine	Fibrillarin	Unknown	[154]
		Lys + 14.0157	S-adenosylmethionine	KMTs	KDMs, JMJDs, LSD1	[155]
		Met + 14.0157	S-adenosylmethionine	SETD3	Unknown	[156]
	asymmetricdimethyl	Arg + 28.0314	S-adenosylmethionine	Type I PRMT	KDM3A^1^, KDM4A^1^, KDM6B^1^, KDM5C^1^	[148-153]
	symmetricdimethyl	Arg + 28.0314	S-adenosylmethionine	Type II, III PRMTs	JMJD6^1^, KDM4E, KDM5C	[148-153]
	1-methyl	His + 14.0157	S-adenosylmethionine	METTL9	Unknown	[157]
	3-methyl	His + 14.0157	S-adenosylmethionine	SETD3, METTL18, HPM1	Unknown	[158]
	dimethyl	Lys + 28.0314	S-adenosylmethionine	KMTs	KDMs, JMJDs, LSD1	[155]
	trimethyl	Lys + 42.0471	S-adenosylmethionine	KMTs	KDMs, JMJDs, LSD1	[155]
**Glycation**	3-DG-H	Arg + 144.0422	3-deoxyglucosone	Non-enzymatic	Unknown	[159]
	3-DGArg	Arg + 162.0528	3-deoxyglucosone	Non-enzymatic	Unknown	[159]
	glyoxal hydroimidazolone	Arg + 39.9949	Glyoxal	Non-enzymatic	Unknown	[160]
	methylglyoxal hydroimidazolone	Arg + 54.0106	Methylglyoxal	Non-enzymatic	DJ-1^1^	[17, 20, 161]
	carboxymethyl	Arg + 58.0055	Glyoxal	Non-enzymatic	Unknown	[160]
		Lys + 58.0055	Glyoxal, 3-deoxyglucosone	Non-enzymatic	Unknown	[160]
	carboxyethyl	Arg + 72.0212	Methylglyoxal	Non-enzymatic	DJ-1^1^	[17, 20, 161]
		Lys + 72.0212	Methylglyoxal	Non-enzymatic	DJ-1^1^	[16, 17, 20]
	argpyrimidine	Arg + 84.0575	Methylglyoxal	Non-enzymatic	Unknown	[161]
	tetrahydropyrimidine	Arg + 144.0422	Methylglyoxal	Non-enzymatic	Unknown	[161, 162]
	pyrraline	Lys + 110.0368	3-deoxyglucosone	Non-enzymatic	Unknown	[159]
	ribosyl	Lys + 132.0423	Ribose	Non-enzymatic	DJ-1^1^, FN3K^1^	[163]
	fructosyl	Lys + 162.0528	Glucose	Non-enzymatic	FN3K^1^	[164]
	glucosyl	Lys + 162.0528	Fructose	Non-enzymatic	FN3K^1^	^2^
	phosphoglyceryl	Lys + 167.9824	3-phosphoglycerate	Non-enzymatic	DJ-1^1^, FN3K^1^	[19]
**Phosphorylation**	phosphorylation	Arg + 79.9663	ATP	Unknown	Unknown	[165]
		Ser + 79.9663	ATP	Kinases	Phosphatases	[166, 167]
		Thr + 79.9663	ATP	Kinases	Phosphatases	[166, 167]
		Tyr + 79.9663	ATP	Kinases	Phosphatases	[166, 167]
	1-phosphoHis	His + 79.9663	ATP	NME1/2	PHPT1, LHPP, PP1/2A/2C, PGAM5	[168, 169]
	3-phosphoHis	His + 79.9663	ATP	NME1/2	PHPT1, LHPP, PP1/2A/2C, PGAM5	[168, 169]
	pyrophosphorylation	Ser + 159.9327	ATP; inositol pyrophosphate diphosphoinositol pentakisphosphate	Multi-step: Kinase 'priming' and subsequent non-enzymatic	Unknown	[170-172]
		Thr + 159.9327	ATP; inositol pyrophosphate diphosphoinositol pentakisphosphate	Multi-step: Kinase 'priming' and subsequent non-enzymatic	Unknown	[170-172]
	polyphosphorylation	Lys + (79.9663)_n_	Inorganic polyphosphate	Non-enzymatic	Endo/exopolyphosphatases	[173, 174]
**Oxidation**	hydroxy	Asn + 15.9949	2-oxoglutarate	Asparaginyl hydroxylase	Unknown	[175]
	citrullination	Arg + 0.9840	H_2_O	Protein Arginine Deiminases (PADs)	Unknown	[176]
	*S*-sulfenyl	Cys + 15.9949	Reactive oxygen/nitrogen species	Non-enzymatic	Reversible, Thioredoxins	[41]
	*S*-sulfinyl	Cys + 31.9898	Reactive oxygen/nitrogen species	Non-enzymatic	Sulfiredoxins	[41]
	*S*-sulfonyl	Cys + 47.9847	Reactive oxygen/nitrogen species	Non-enzymatic	Irreversible	[41]
	*S*-nitrosyl	Cys + 28.9901	Reactive nitrogen species, S-Nitrosoglutathione	Non-enzymatic	Thioredoxin, GSH; Serves as an intermediate to disufide bond generation	[177, 178]
	*S*-succination	Cys + 116.0109	Fumarate	Non-enzymatic	Unknown	[179]
	*S*-itaconation	Cys + 130.0267	Itaconate	Non-enzymatic	Unknown	[89]
	*S*-cysteinyl	Cys + 119.0041	Cysteine	Non-enzymatic	Reversible	[180, 181]
	*S*-homocysteinyl	Cys + 133.0198	Homocysteine	Non-enzymatic	Reversible	[181]
	*S*-glutathionyl	Cys + 305.0681	GSSG, GSNO, GSH	Glutathione S-transferases, Non-Enzymatic	Reversible, Thioredoxins, Glutaredoxins	[182]
	5-hydroxylation	Lys + 15.9949	2-oxoglutarate	JMJD6, lysyl hydroxylases	Unknown	[183, 184]
	allysyl	Lys - 1.0316	O_2_, H_2_O	Lysyl oxidases	Unknown	[185]
	carbamyl/homocitrullination	Lys + 43.0058	OCN^-^	Non-enzymatic	Unknown	[186]
	carboxyl	Lys + 43.9898	CO_2_	Non-enzymatic	Reversible	[186]
		Glu + 43.9898	Vitamin K, CO_2_, O_2_	γ-glutamyl carboxylase	Unknown	[187, 188]
	homocysteinyl	Lys + 174.0460	Homocysteine thiolactone	Non-enzymatic	Unknown	[189]
	oxidation	Met + 15.9949	Reactive oxygen species	Non-enzymatic, Methionine sulfoxide reductases	Methionine sulfoxide reductases	[190]
	dioxidation	Met + 31.9899	Reactive oxygen species	Non-enzymatic	Unknown	[191]
	hydroxy	Pro + 15.9949	2-oxoglutarate	Prolyl hydroxylase	Unknown	[192]
	4-HNE	Cys + 156.1151	4-hydroxy-2-nonenal	Non-enzymatic	Unknown	[193]
		His + 156.1151	4-hydroxy-2-nonenal	Non-enzymatic	Unknown	[193]
		Lys + 140.1201	4-hydroxy-2-nonenal	Non-enzymatic	Unknown	[193]
		Lys + 156.1151	4-hydroxy-2-nonenal	Non-enzymatic	Unknown	[193]
	ketoamide	Lys + 154.0994	4-oxo-2-nonenal	Non-enzymatic	SIRT2	[194-196]
	MDA	Lys + 54.0106	Malondialdehyde	Non-enzymatic	Unknown	[197]
		Lys + 134.0368	Malondialdehyde	Non-enzymatic	Unknown	[197]
	*N*-formylkynurenine	Trp + 31.9899	Reactive oxygen species	Non-enzymatic	Unknown	[198]
	kynurenine	Trp + 3.9949	Reactive oxygen species	Non-enzymatic	Unknown	[198]
	3-hydroxy	Tyr + 15.9949	Reactive oxygen species	Non-enzymatic	Unknown	[199]
	*O*-sulfo	Tyr + 79.9568	3′-phosphoadenosine 5′-phosphosulfate	Tyrosyl-protein sulfotransferase	Unknown	[200]
	3-nitroTyr	Tyr + 44.9851	Reactive nitrogen species	Non-enzymatic	Unknown	[199]
**Acylation**	*S*-acetyl	Cys + 42.0106	Acetyl-CoA, Acetyl-GSH	Non-enzymatic	Unknown	[18]
	*S*-palmitoyl	Cys + 238.2297	Palmitoyl-CoA	DHHC domain proteins, Non-enzymatic	Acyl-protein thioesterases, Non-enzymatic hydrolysis	[102, 201]
	*S*-oleoyl	Cys + 266.2610	Oleoyl-CoA	DHHC domain proteins, Non-enzymatic	Acyl-protein thioesterases, Non-enzymatic hydrolysis	[102, 202]
	*S*-stearoyl	Cys + 266.2610	Stearoyl-CoA	DHHC domain proteins, Non-enzymatic	Acyl-protein thioesterases	[102, 201, 202]
	formyl	Lys + 27.9949	Formylphosphate, 10-formyl-tetrahydrofolate^2^	Non-enzymatic	HDAC1/2/3, SIRT3/6	[63, 203, 204]
	acetyl	Lys + 42.0106	Acetyl-CoA; Acetyl-GSH	KATs, Gcn5, p300/CBP, PCAF, NatA, Tip60, MOF; Non-Enzymatic	KDACs, SIRTs	[117, 205]
	propionyl	Lys + 56.0262	Propionyl-CoA	p300/CBP, GNATs, MYSTs, HAT1, Non-Enzymatic	HDAC1/2/3, SIRT1/2/3	[64, 206-208]
	crotonyl	Lys + 68.0262	Crotonyl-CoA	p300/CBP, GNATs, MYSTs, Non-enzymatic	SIRT1/2/3, HDAC1/2/3	[61, 206, 209, 210]
	butyryl	Lys + 70.0419	Butyryl-CoA	Non-enzymatic, GNATs	SIRT1/2/3	[64, 206, 207, 211]
	isobutyryl	Lys + 70.0419	Isobutyryl-CoA	p300	HDAC1/2/3, SIRT2	[208, 209, 212]
	lactoyl	Lys + 72.0212	lactyl-CoA^2^; lactoylGSH	p300^2^; Non-ezymatic	HDAC1/2/3 and SIRT2	[16, 44, 95]
	tiglyl	Lys + 82.0419	Tiglyl-CoA	Non-enzymatic	Unknown	[213]
	acetoacetyl	Lys + 84.0211	Acetoacetyl-CoA	Non-enzymatic	Unknown	[213]
	2-methylbutyryl	Lys + 84.0575	2-methylbutyryl-CoA	Unknown	Unknown	[213]
	isovaleryl^2^	Lys + 84.0575	isovaleryl-CoA	Unknown, Non-enzymatic	HDAC1/3	[209, 214]
	malonyl	Lys + 86.0004	Malonyl-CoA	KAT2A	SIRT5	[141, 215]
	2-hydroxybutyryl	Lys + 86.0368	β-hydroxybutyryl-CoA	p300	HDAC1/2/3, SIRT1/2	[119, 145, 146, 216]
	β-hydroxyutyryl	Lys + 86.0368	β-hydroxybutyryl-CoA	p300	HDAC1/2/3, SIRT1/2	[146]
	hexanoyl	Lys + 98.0732	Hexanoyl-CoA	Unknown, Non-enzymatic	HDAC1/2/3/11, SIRT1/2/3/6	[203, 217]
	succinyl	Lys + 100.0160	Succinyl-CoA	CPT1a	SIRT5	[211, 218-220]
	benzoyl	Lys + 104.0262	Sodium benzoate	Lys acyltransferase^2^	SIRT2	[92]
	glutaryl	Lys + 114.0317	Glutaryl-CoA	Unknown, Non-enzymatic	SIRT4/5	[211, 221]
	3-methylglutaconyl	Lys + 126.0317	Methylglutaconyl-CoA	Unknown, Non-enzymatic	SIRT4	[221]
	octanoyl	Lys + 126.1045	Octanoyl-CoA	Unknown, Non-enzymatic	SIRT6, HDAC8/11	[102, 197, 217, 222, 223]
	3-methylglutaryl	Lys + 128.0473	Methylglutayryl-CoA	Unknown, Non-enzymatic	SIRT4	[211, 221]
	3-hydroxy-3-methylglutaryl	Lys + 144.0423	HMG-CoA	Unknown, Non-enzymatic	SIRT4	[211, 221]
	decanoyl	Lys + 154.1358	Decanoyl-CoA	Unknown, Non-enzymatic	SIRT6/7, HDAC1/2/3/11	[208, 209, 217]
	dodecanoyl	Lys + 182.1671	Dodecanoyl-CoA	Unknown, Non-enzymatic	HDAC8/11	[217, 223]
	lipoyl	Lys + 188.0330	Multi-step, involves octanoyl-acyl carrier protein	Multi-step: LIPT1, LIPT2, LIAS, glycine cleavage H protein.	HDAC11, SIRT2/4	[197, 217, 224, 225]
	myristoyl	Lys + 210.1984	Myristoyl-CoA	N-terminal Gly myristoyltransferases 1 and 2	SIRT2/6, HDAC1/2/8/11	[102, 217, 222] [209, 223, 226, 227]
	N_6_-(pyridoxalphosphate)	Lys + 229.0140	Pyrixoxal 5'-phosphate	Non-enzymatic	Unknown	[228]
	palmitoyl	Lys + 238.2297	Palmitoyl-CoA	Hedgehog acyltransferase	SIRT6, HDAC11	[102, 217, 222]
	*O*-acetyl	Ser + 42.0106	Acetyl-CoA	Unknown	Unknown	[229, 230]
	*O*-octanoyl	Ser + 126.1045	Octanoyl-CoA	Ghrelin *O*-acyltransferase	Butyrylcholinesterase, Acyl-protein thioesterase 1, α2-macroglobulin, Notum	[231]
	*O*-myristoyl	Ser + 210.1984	Myristate	Unknown	Unknown	[232]
	*O*-palmitoyl	Ser + 238.2297	Palmitoyl-CoA	Membrane-bound O-acyl-transferases	Notum	[102]
	*O*-acetyl	Thr + 42.0106	Acetyl-CoA	Unknown	Unknown	[229, 233]
	*O*-myristoyl	Thr + 201.1984	Myristate	Unknown	Unknown	[232]
	*O*-palmitoyl	Thr + 238.2297	Palmitoyl-CoA	Membrane-bound O-acyl-transferases	Notum	[102]
	*O*-acetyl	Tyr + 42.0106	Acetyl-CoA	Unknown	Unknown	[229, 234]
**Lipidation**	farnesylCys	Cys + 204.1878	Farnesyl diphosphate	Farensyl transferase	Esterases^2^	[102]
	geranylgeranylCys	Cys + 272.2504	Geranylgeranyl diphosphate	Geranylgeranyl transferase; Rab geranylgeranyl transferase	Esterases^2^	[102]
**Other**	*N*-glycosylation	Asn + Variable	*N*-acetylglucosamine	Oligosaccharyltransferases	PNGase/N-glycanase	[235]
	*O*-glycosylation	Ser + Variable	*N*-acetylgalactosamine, N-acetylglucosamine, dolichol-*P*-mannose, fucose, glucose	O-GlcNAc transferase, O-glucosyltransferases	O-GlcNAcase	[235, 236]
		Thr + Variable	*N*-acetylgalactosamine, N-acetylglucosamine, dolichol-*P*-mannose, fucose, glucose	O-GlcNAc transferase, O-glucosyltransferases	*O*-GlcNAcase	[235, 236]
		Tyr + Variable	*N*-acetylgalactosamine	O-GlcNAc transferase	Unknown	[197, 237]
	*S*-guanyl	Cys + 343.0318	8-nitroguanosine 3',5'-cyclic monophosphate	Non-enzymatic	Unknown	[238]
	*S*-sulfhydryl	Cys + 31.9720	Hydrogen sulfide	Non-enzymatic	Reversible	[239]
	CoAlation	Cys + 765.0995	Coenzyme A	Non-enzymatic	Reversible	[240]
	histaminyl	Gln + 94.0531	Histamine	Transglutaminase 2	Unknown	[241]
	dopaminyl	Gln + 136.0524	Dopamine	Transglutaminase 2	Unknown	[242]
	serotonyl	Gln + 159.0685	Serotonin	Transglutaminase 2	Unknown	[243]
	biotinyl	Lys + 226.0776	Biotin	Holocarboxylase synthetase	HDAC11, SIRT4	[217, 244, 245]
	hypusination	Lys + 87.0684	Spermidine	Multi-step process: deoxyhypusine synthase, deoxyhypusine hydroxylase	Unknown	[246, 247]
	ubiquityl	Lys + 114.04293^3^	Ubiquitin	Multi-step process involving E1, E2, and E3 ubiquitin ligases	Deubiquitination enzymes (Dubs)	[47, 48, 197, 248]
	NEDDyl	Lys + 114.04293^3^	NEDD	Multi-step process involving NAE, UBC12/UBE2F, and E3 ubiquitin ligases	COP9 signalosome, deneddylase 1	[48, 249]
	SUMOyl	Lys + 114.04293^3^	SUMO	Multi-step process involving SAE1 or SAE2, Ubc9, and E3 SUMO ligases	Serine specific proteases (SENPs)	[48, 197, 250, 251]
	C-linked glycosylation	Trp + Variable	α-D-mannopyranose	*C*-Man glycosyltransferases	Unknown	[235, 236, 252, 253]
	3-chloro	Tyr + 33.9610	HOCl	Non-enzymatic	Unknown	[254]
	ADPRibosylation	Arg + 539.0466	ADP-ribose	ADP-ribosyltransferases	Mono-ADP-ribosylhydrolases	[197]
		Cys + 539.0466	ADP-ribose	ADP-ribosyltransferases	Mono-ADP-ribosylhydrolases	[197]
		Glu + 539.0466	ADP-ribose	ADP-ribosyltransferases	Mono-ADP-ribosylhydrolases	[197]
		Lys + 539.0466	ADP-ribose	ADP-ribosyltransferases	Mono-ADP-ribosylhydrolases	[197]
		Ser + 539.0466	ADP-ribose	ADP-ribosyltransferases	Mono-ADP-ribosylhydrolases	[197]
		Thr + 539.0466	ADP-ribose	ADP-ribosyltransferases	Mono-ADP-ribosylhydrolases	[197]
	PolyADPRibosylation	Variable	ADP-ribose	Poly(ADP-ribosyl)polymerases	Poly-ADP-ribose Glycohydrolases	[197]

1 Under debate;

2 Speculated;

3 Following tryptic digestion;

Most Lys acylations can be derived through non-enzymatic means; however, this does not exclude the possibility of a yet-to-be identified writer.

In the context of cell fate, there is no place where the regulation of PTMs is more critical than the nucleus [7]. Histone PTMs regulate chromatin structure, DNA accessibility, and serve as molecular handles for transcriptional machinery [8,9]. Over the past decade, advances in the sensitivity and specificity of mass spectrometry (MS) and DNA sequencing have led to a significant expansion in the number of functionally relevant histone PTMs [10]. To date, >100 types of PTMs have been identified on histones, many of which play a critical role in regulating transcriptional responses to endogenous and xenobiotic stimuli [11,12]. Given the consequences of a misplaced or the prolonged occupancy of a PTM, many are strategically placed onto target residues through ‘writers’ (e.g. kinases) and removed via ‘erasers’ (e.g. phosphatases) [13]. PTMs also serve as molecular handles for transcriptional machinery, providing a scaffold for ‘reader’ domain recognition (e.g. 14-3-3 domains) [13]. As many of these processes go awry in disease, significant effort has been placed on targeting the PTM landscape in chromatin, with numerous drugs currently on the market and/or making their way through clinical trials [14].

While many PTMs are tightly regulated through enzyme-catalyzed reactions, there is now mounting evidence that PTMs generated through non-enzymatic means may play a significant role in cell signaling, rather than serving as simple markers of toxicity [15–20]. A prime example of this is Lys acetylation, which is heavily enriched on glycolytic and TCA pathway enzymes, controlling metabolic flux and maintaining homeostasis [21]. Although Lys acyltransferases (e.g. p300) are established writers of these marks, the bulk of non-nuclear Lys acetylation is now postulated to arise from a non-enzymatic, S-to-N acyl-transfer from acetyl-CoA to an unmodified Lys residue [18]. This does classify these PTMs are unregulated, however, as Lys deacylases and sirtuins (SIRTs) remove modifications located at residues critical for function [22–25]. With >2 000 000 PTM sites now experimentally validated [26], one has to ask: *Are all PTMs regulatory?*

In this review, we will highlight the field of PTM research from the proverbial 30 000-foot view, solely focusing on modifications to amino acid sidechains and neglecting N- and C-terminal modifications. As an in-depth review could be written about nearly every PTM, our intent is not to take a deep dive into any single modification, but rather take a global view the field, the mechanisms that govern PTM site-specificity, and the technologies to investigate their role in health and disease. We will also close by discussing a growing notion that many PTMs simply serve as a metabolic reserve for nutrients and play a minimal role in the regulation of protein function. This review also aims to serve as a starting point and resource for those interested in studying PTMs using thorough analytical approaches.

## PTMs are diverse and dynamically regulated

Although nearly every amino acid is prone to some degree of post-translational processing, the majority of reported PTMs reside on the nucleophilic side-chains of Lys, Arg, Cys, Ser, Thr, and Tyr [26] (Figure 1). As every protein contains amino acids with modifiable sidechains, it should not be surprising that nearly every protein has been reported, or predicted, to contain at least one PTM. Given the diversity in PTM composition and protein targets, a complete survey of the PTM landscape in biology is a seemingly insurmountable task; however, bioinformatic approaches have provided considerable insight into their prevalence [26,27]. dbPTM (https://awi.cuhk.edu.cn/dbPTM/) is an open access software that mines and curates existing PTM databases to provide functional and structural insights into >2 000 000 experimentally validated PTM sites [28]. This should be interpreted with some caution, however, as the prevalence of a select few PTMs (e.g. Ser/Thr phosphorylation, which accounts for >70% of experimentally validated PTM sites) are likely skewed due a strong focus on these pathways in biomedical research and ready access to tools/resources (i.e. phospho-‘specific' antibodies) [28]. While the importance of phosphorylation should not be understated, comprehensive studies evaluating its abundance have revealed a more modest estimation of ∼80 000 sites, with the nearly twice as many found to result from false-positive identifications [29].

Many PTMs are derived from primary metabolic intermediates and serve as sensors for nutrient status in the cell [6,7,30]. Acylations, for example, are a class of structurally diverse PTMs that exist on Lys, Ser, Thr, and Cys [31]. These PTMs range from a simple acetylation (C_2_H_3_O) to more complex modifications such as palmitoylation (C_16_H_31_O) (Figure 1) [6]. The bulk of acylations are derived from their parent acyl-CoA species and may be enzymatically or non-enzymatically placed onto proteins [30]. As a result, the abundance of protein acylation is largely reflective of local acyl-CoA concentrations [32,33]. In addition, acyl-GSH conjugates have also been identified as a major contributor to the acylLys pool, solely through a similar non-enzymatic S-to-N acyl transfer [16,18,34]; the contribution of acyl-CoA vs acyl-GSH towards the PTM pool, however, is unknown. Regardless of the route of addition, Lys acylations are dynamically regulated through their enzymatic removal via Lys deacylases and SIRTs, displaying half-lives ranging from minutes to >150 h [35]. These highly variable half-lives also differ among sites on the same protein, suggesting more active regulation of potentially regulatory sites, while allowing agnostic sites to maintain their occupancy [35]. Perhaps unsurprisingly, proteins involved with transcription/translation generally display shorter half-lives of acetylation sites, with many histones acetylation sites having half-lives ≤1 h [36]. Histone proteins have exceptionally long half-lives, with some primary cell lines displaying an average half-life on the order of weeks [37]. PTM status drastically impacts histone half-life, with Lys methylations (generally associated with heterochromatic, silenced regions of the genome) reducing histone turnover and acetylation (generally associated with euchromatic regions of the genome) increasing histone turnover [38]. While much work has been done in the field of acetylation, little is known about the relative half-lives of other Lys acylations and their impact on protein turnover.

Non-enzymatic PTMs are vital components to cell signaling and protein function, despite the long-held belief that they solely serve as markers of cell toxicity [39]. A prime example of this is Cys oxidation, which was originally used as an indication of oxidative stress and protein damage [40]. Cys oxidation is necessary for a myriad of biological processes, ranging from protein folding to transcriptional responses to stress [40]. Accumulation of these PTMs above the hormetic, or adaptive, threshold, however, is now appreciated as the critical determinant in establishing toxic responses [40]. Although a comprehensive investigation into Cys oxidation half-life has not been completed, estimations suggest sulfenylation, for example, is rapidly turned over, with half-lives <5 min [41]. Elegant studies have demonstrated that this quick turnover serves a regulatory role in metabolic flux, slowing output during periods of nutrient excess. GAPDH, a primary glycolytic enzyme, shows a near complete loss of activity <5 min after exposure to H_2_O_2_ due to the oxidation of an active site Cys and full restoration 30 min post-exposure [42]. This aligns with proposed role of Cys in biology, where up to 80% of Cys residues are predicted to be ‘functional’ [42,43].

Protein glycation has taken a similar path to Cys oxidation, long believed to be a measure of glucose toxicity and routinely used as a biomarker for glycemic control (i.e. HbA1c). This complex class of PTMs predominately reside on Arg and Lys, existing at concentrations comparable to other canonical, enzymatically regulated, protein modifications (e.g. asymmetric dimethylArg) [17]. Despite their non-enzymatic origins, protein glycation now has defined roles in the regulation of chromatin and metabolic flux [16,17,20]. Lys lactoylation, which is derived from the glycolytic by-products lactoylglutathione and lactate, are enriched on primary glycolytic enzymes, reducing metabolic output and slowing glycolytic flux [16,44]. These findings are critical in establishing a functional role for non-enzymatically generated PTMs, which should not classify a PTM as damaging or toxic to cell health but rather, important for maintaining homeostasis. Of course, like most other PTMs, the threshold dictating a homeostatic function from a toxic response (i.e. cell death) likely resides in its abundance [20,45]. Often, studies linking protein glycation to toxic responses rely on supraphysiological concentrations of glycating agents and thus careful consideration must be taken when employing model systems to explore the physiological role of non-enzymatic PTMs, such as glycation.

While small molecule modifications comprise the bulk of PTMs in biology, protein conjugates also have a critical role in cell biology. Lys ubiquitylation is an evolutionarily conserved PTM that regulates a multitude of biological processes, most notably protein degradation and turnover [46]. Ubiquitin is a 76 amino acid protein that is conjugated via an isopeptide bond with its C-terminal Gly carboxylate and a target Lys ε-amino group [47]. Akin to ubiquitylation, SUMOylation and NEDDylation are small ubiquitin-like protein modifications that are indispensable in eukaryotes [48]. These PTMs are enzymatically placed at precise Lys residues through a multi-step enzymatic process and, despite the structural and functional similarities in these three PTMs, the sites of modification vary widely; ubiquitylation is the most abundant (>60 000 sites), with SUMOylation (∼8000 sites) and NEDDylation (∼1000 sites) following [48]. Importantly, little overlap in target sites exists (∼100) between these three PTMs, suggesting functional independence despite the similarities in signaling and regulation [48]. What makes these PTMs particularly unique is their ability to undergo post-translational modifications themselves, through either or small molecule modifications (e.g. phosphorylation) or extensive homo- or hetero-poly-ubiquitin/NEDD/SUMO branching [48–50].

PTMs rarely exist in isolation as proteins are prone to undergo a wide array of diverse modifications simultaneously at numerous sites. Many PTMs trigger, or prime, proteins to undergo additional modification, which elicit alterations in function and/or localization [51,52]. This ‘PTM crosstalk’ is perhaps most well appreciated on histones where PTMs influence the binding of writers and erasers resulting in subsequent histone modifications [53]. For example, asymmetric dimethylation of Arg2 on histone H3 serves as a transcriptional repressive mark at promoters by preventing Lys methyltransferase 2A from methylating Lys4 of histone H3 [54,55]. Conversely, symmetric dimethylation of H3 Arg2 promotes trimethylation at Lys4 through binding of WDR5, a common component of coactivator complexes and euchromatic regions of the genome [54,55]. This is just one example of numerous instances of PTM crosstalk that have been elegantly defined in biology and certainly, this layering of PTMs adds additional layers of complexity in the study of PTMs [52,56].

## Methods for the detection and study of PTMs

### Antibody-based approaches

Antibody-based approaches remain a staple in PTM research, largely due to the ubiquity and relative ease of immunoassays. Pan-antibodies that broadly recognize a PTM, regardless of the target protein (e.g. anti-acetylLys) are useful tools to determine relative changes across the proteome in a set of biological samples. These antibodies are also routinely used for co-immunoprecipitations to investigate alterations on target proteins. It should be noted, however, that these approaches are constrained to the analysis of a single PTM, failing to provide information on its abundance or stoichiometry. In addition, poorly vetted and low-fidelity antibodies have polluted PTM research leading to false assertions as to the role of these modifications in biology and disease. Pertaining to histone PTMs, thorough investigations have revealed that >25% of all antibodies failed specificity tests [57,58]. Combinatorial PTMs also disrupt antibody specificity, whereby occupancy of nearby residues with PTMs can alter epitope recognition [57,58]. This is particularly relevant for histones, where the N-terminal tails are heavily modified with a diverse array of PTMs. While there are significant drawbacks to antibody-based approaches, they have no doubt transformed our ability to detect, enrich, and study PTMs on a broad scale. It is important moving forward, however, that antibodies are properly validated (i.e. knockout cell lines) and coupled to additional means of specificity (e.g. mass spectrometry, site-directed mutagenesis).

### MS-based approaches

**Figure 2.**
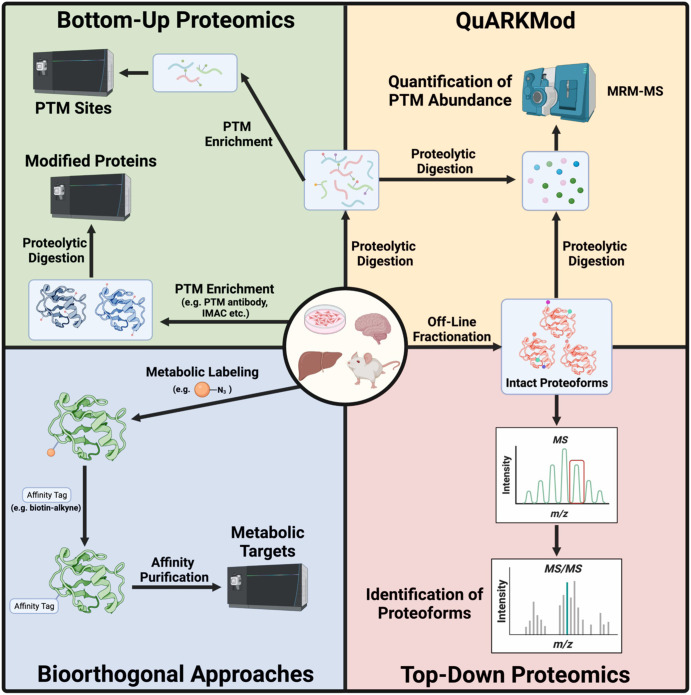
Analytical approaches for the study of PTMs. Created with BioRender.com.

The past two decades have seen a rapid rise in both sensitivity and accuracy of mass spectrometers (MS) [59]. This has undoubtedly fueled the rapid expansion in the list of known PTMs in biology [28]. Although numerous MS-based approaches can be applied for PTM research, bottom-up proteomics remains the most prevalent and logical starting point. Here, proteins are proteolytically digested to peptides, separated via liquid-chromatography (LC), and analyzed using tandem mass spectrometry (MS/MS) for PTMs of interest. As PTMs are typically low in abundance compared with their unmodified counterparts, sample enrichment is almost always necessary to gain a full picture of the PTM profile [60]. By far the most common enrichment strategy is through immunoaffinity columns, using antibodies directed at either the protein or PTM of interest (Figure 2). In addition to antibody-based approaches, numerous off-line enrichment strategies can be employed: isoelectric focusing and SDS–PAGE can reduce proteome complexity, with the goal of isolating the protein(s) of interest [61–64]. Multi-dimensional chromatography approaches, such as high-pH [65,66], size exclusion [67], and strong cation exchange chromatography [68,69] have been widely utilized to increase resolution and dynamic range. When coupled with MS, these platforms can be used to generate high quality, accurate mass identification, quantification, and discovery of PTMs (Figure 2). This approach provides considerable flexibility in sample preparation, LC conditions, and ionization strategies; however, the most common proteomic workflow uses a tryptic digestion and reversed-phase LC coupled to an electrospray ionization MS. Trypsin, which cleaves after Lys and Arg, is a high-fidelity enzyme, yielding consistent peptides and reproducible MS data. There are significant caveats to this approach, however; tryptic digestion may not provide coverage across a desired site of modification, yielding peptides too large for MS detection (often <1500 *m/z*). A simple solution to this problem is to use an alternative protease (e.g. chymotrypsin, Glu-C), yielding peptides better suited for MS detection that span the site of interest . Conversely, proteins rich in Lys or Arg (e.g. histones) may yield tryptic peptides too short or polar for detection with standard reversed-phase LC conditions [70]. To address this, chemical derivatization strategies (e.g. propionylation) can be used to modify free Lys residues [70]. This propionylated Lys residue will result in a missed cleavage, leading to cleavage at the next available unmodified Arg (Figure 2). This strategy also increases the hydrophobicity of smaller peptides, resulting in increased retention on standard reversed-phase LC systems and improved MS detection [70]. A caveat to this approach, however, is the inability to differentiate endogenous vs. exogenous propionylation. To circumvent this problem, isotopically labeled propionic anhydride (e.g. d_10_) can be used during chemical derivitization [71].

As noted above, PTMs are often low in abundance and challenging to detect in crude proteome preparations using MS; thus, sample enrichment is typically required. This is most commonly achieved through immunoaffinity columns using proteolytically digested samples, selectively isolating only peptides containing the PTM of interest [72]. As unmodified peptides are lost during immunoprecipitation, reference proteome samples collected prior to enrichment are typically run in parallel using label-free quantitation approaches (i.e. spectral counting) [73]. Despite this experimental simplicity and adaptability to analyze large datasets, bias in sample preparation and data processing may hinder accuracy, precision, and reproducibility [73]. To circumvent this problem, numerous labeling approaches may be employed. Stable Isotope Labeling by Amino acids in Cell culture (SILAC) [74], chemical labeling using isobaric tags (i.e. iTRAQ) [75], and tandem mass tags [76] are routinely employed prior to immunoprecipitation. This provides a measure of relative quantitation in the form of heavy isotope to light isotope ratios. While these approaches provide relative quantification of proteins in a given dataset, stable isotope labeled protein or peptide standards can alternatively be spiked in prior to immunoprecipitation, yielding absolute quantification or a target protein/PTM [72].

Another popular strategy for the quantitation of PTMs in biological samples relies on multiple reaction monitoring (MRM)-MS [77]. Here, target sites and fragment ions are known and peptide masses are included in the MS parameters [77]. This results in increased sensitivity and provides improved sequence coverage to ensure that all sites are mapped [77]. From here, two strategies can be used for quantitation: (1) all modified peptide masses from a single site are summed and set as the denominator, with the target PTM set as the numerator, providing the relative abundance of a PTM at a given site [78]. With this approach, MS parameters must be optimized for each peptide as many PTMs are known to alter ionization efficiency thus biasing their abundance [78,79]; and (2) synthetic isotopically labeled peptides standards can be added for absolute quantification [77]. These approaches are routinely used for the analysis of histone PTMs due to the high degree of modification states and ease of histone isolations from complex biological samples, reducing interference from non-histone proteins. It should also be noted that these quantitation strategies are also amenable to standard shotgun proteomic approaches and are not unique to the MRM-MS platform.

The heterogeneity of peptides makes the discovery of PTMs a significant challenge, and while database algorithms have provided some hope (see below), these approaches are not overly amenable to most labs. To remove the complexity of peptides from the equation, QuARKMod, or the Quantitative Analysis of Arg (R) and Lys (K) Modifications (QuARKMod), relies on exhaustive proteolytic digestion, yielding single amino acids (Figure 2) [60]. Amino acids are then separated and detected using an MRM approach on a triple quadrupole MS. A major advantage to this approach is the increased sensitivity and, provided stable isotope standards are included, determination of PTM abundance, allowing for a comparison of all measured PTMs in a given sample [60]. The chromatographic separation of single amino acids also provides improved separation of isomeric species compared with that of peptides. This was demonstrated through the identification of lactoylLys, which is isomeric to the advanced glycation end-product, carboxyethylLys [16]. In addition, this method can be applied for PTM discovery using a precursor-ion scan for Lys or Arg fragments (84.1 *m/z* and 70.1 *m/z*, respectively) that remain consistent, regardless of the PTM [60]. While this approach provides an accurate measure of PTM abundance, this comes at the expense of site identification as all sequence information is lost following proteolytic digestion.

Undoubtedly the utility of bottom-up proteomic approaches has been instrumental in the discovery and quantitation of PTMs in biology. A significant shortcoming of these approaches, however, is that PTM stoichiometry on a single molecule is lost. Top-down proteomic approaches have aimed to address this, by ionizing intact proteins and analyzing subsequent fragment ions [80]. This yields complete sequence coverage, improves isoform resolution, and provides a comprehensive view of the PTM profile and interplay on a target protein (i.e. proteoforms) [81]. This was elegantly displayed with KRAS4b, one of four isoforms in the RAS family, which share high sequence homology (82–90%) [82]. KRAS4b undergoes post-translational processing of a conserved C-terminal CaaX motif, ultimately yielding carboxymethylation to the C-terminal Cys residue. This modification is essential for association with the plasma membrane and interaction with effector proteins. Typical bottom-up approaches yield identical peptides from each RAS isoform, making isoform-specific analysis an insurmountable task. Using top-down proteomics, 11 proteoforms of KRAS4b can be resolved, revealing a high degree of variability among cancer patients in this critical C-terminal processing [81]. Although the advantages to this approach are clear, there are significant hurdles preventing the mainstream application of top-down proteomics: cumbersome prefractionation of intact proteins, lower sensitivity compared with MRM approaches, limitations in protein size (<100 kDa), intact protein solubility (particularly with membrane proteins), the need for high-resolution mass analyzers, and complexity of data analysis [83,84].

### Database algorithms for PTM identification

Due to the chemical heterogeneity of PTMs, MS and MS/MS spectra are often complex [62]. Database algorithms are thus a crucial component in the PTM workflow, mining spectra for PTMs of interest. Software such as X!Tandem [85] and Mascot [86] provide the user with the ability to search datasets for defined mass shifts on target residues of both known and unknown PTMs (Table 1). A major hinderance to these software packages is the limited number of variable modifications (i.e. PTMs) that can be searched in a single analysis, with each PTM resulting in an exponential rise in false-positive identifications. While data analysis software has become quite robust in recent years (<1% false discovery rate), it is still imperative to manually validate sites of interest, particularly for novel or poorly characterized PTMs. Separate software packages have also been developed to try and reduce the manual mining of MS spectra: PILOT_PTM can search an unrestricted number of PTMs on a template sequence (i.e. known site) [87]. Spectral Alignment-based Modified Peptide Identification, or SAMPEI, was developed based on the rationale that MS/MS spectra of protein are produced from both modified and unmodified peptides, and thus any statistics-based algorithm, such as X!Tandem, can be used for peptide sequence matches (PSMs) [88]. The remaining unmatched MS/MS spectra are used as target spectrum to identify peptide sequences with PTMs. Using a cellular model for inflammation, SAMPEI identified a mass shift corresponding to Cys +130.03 Da and Cys +146.02 Da, consistent with Cys itaconatylation and an oxidized product of this PTM, respectively [88]. These PTMs have known and appreciated roles in inflammatory signaling and have been identified on numerous metabolic enzymes [88,89]. This simple application was able to demonstrate the robustness of SAMPEI in the identification of unknown PTMs. However, since SAMPEI depends on statistical-based algorithms such as X!Tandem, the number of searched PTMs is limited.

Open search, which allows for a larger precursor mass tolerance resulting in the analysis of more spectra, is employed to explore the vast diversity of PTMs that are not accounted for in database search algorithms; however, achieving a comprehensive PTM search with practicality requires a sophisticated computational strategy. MSFragger, generates a theoretical fragment-ion index of peptides from *in silico* digestion of a protein database [90]. Mass binning and precursor mass ordering in the fragment-ion index enables rapid selection of candidate spectra matching experimental fragment ions, allowing all candidate spectra to be simultaneously scored against an experimental spectrum. The algorithm offers advantageous speed in open search compared with conventional database-search algorithms where theoretical spectra of *in silico* digested peptides are compared with experimental spectra. It is noteworthy that an inaccurate estimation of FDR occurs in a narrow-window search using a target-decoy strategy and this can be improved by accounting for all modifications under an open search strategy [90]. In addition, the localization of PTMs further reduces FDR. Some PTMs with identical or similar mass shifts on different amino acids cannot be distinguished without proper localization . PTMiner employs an interactive-leaning based algorithm for the accurate localization of both known and unknown PTMs in open search, which is instrumental in achieving <1% FDR [91].

The sequence alignment software, PTMap, has been immensely successfully in identifying novel PTMs, particulary on histones, including Lys succinylation, Lys crotonylation, Lys benzoylation, and Lys lactylation [61,92–95]. PTMap was the first unrestrictive search algorithm to use unmatched peaks in MS/MS spectra to eliminate false positives while simultaneously using matched peaks to select candidate peptides. To accomplish this, PTMap uses two steps to carry out the localization and putative mass of a PTM: first, iterative comparisons of the MS/MS spectra of experimental peptides to all theoretical MS/MS spectra of the candidate peptide isoforms with all possible PTMs localization. Second, PTMap looks for consecutive y- and b-ions or simultaneously modified y-and b-ions for confident identification of PTM site. While PTMap does not provide any insights into the molecular structure, high resolution mass accuracy does provide a necessary starting point by which hypotheses can be generated to formulate the composition of the unknown PTM.

### Ensuring analytical rigor for increased reproducibility

MS-based proteomic studies have become commonplace in PTM research; however, appropriate analytical rigor must be maintained to ensure reproducibility. While many studies are focused on bulk PTM inventories using antibody-based pulldowns, it is important to manually validate and characterize sites of interest. This can be achieved using synthetic peptides to demonstrate co-elution and identical MS/MS fragmentations (i.e. y- and b-ions) [61,94]. This is particularly important when studying PTMs using low mass accuracy instrumentation (i.e. triple quads). For example, Lys acetylation and Lys trimethylation differ by only 0.0365 Da (Table 1) and are thus indistinguishable on low resolution instrumentation, yet have significant differences in their polarity and can be easily separated using RP-LC [60,77]. The importance of this synthetic peptide approach has been elegantly demonstrated with Lys 2-hydroxyisobutyrylation [96]. The mass shift resulting from this PTM (Lys +86.0368) can be derived from five possible isomers. Using synthetic peptides, co-elution with Lys 2-hydroxyisobutyrylation was demonstrated, showing this to be the dominant species *in vivo* [96]*.* Alternatively, stable isotope labeling can be used to verify PTMs and their metabolic origins. Here, a metabolic precursor (e.g. ^13^C_6_ glucose) is fed to cells and the incorporation of isotopic labels can be monitored on PTMs of interest (e.g. +2 Da for Lys acetylation) [97]. This added layer of confidence also provides the opportunity to monitor PTM half-life and protein turnover. These additional experimental measures increase analytical rigor are necessary to ensure reproducibility within the field.

## Using chemical tools to study PTMs

Although immunoaffinity enrichments are commonplace in PTM research, chemical biology approaches are being adapted at an increasing rate and offer improved specificity and assay diversity. The advent of click chemistry-based probes has transformed our ability to study PTMs in cells with unparalleled specificity. By appending relatively inert bioorthogonal chemical tags (e.g. alkyne/azide) onto metabolic PTM precursors, it is possible to track the incorporation and regulation of these modifications over time, providing information on PTM half-life, protein turnover, and cellular fate (Figure 2). The first application of click chemistry for PTM research was applied for the study of mucin-type O-linked glycosylation [98]. Here, a bioorthogonal monosaccharide bearing an azide or alkyne functional group is fed to cells, resulting in the incorporation of tagged PTMs through the cells natural *N*-acetyl-a-galactosaminyltransferases [98,99]. Click chemistry is then employed using a variety of reporter tags to selectively visualize (e.g. alkynyl/azido fluorophores) or enrich (e.g. alkynyl/azido biotin) samples for modified proteins. As reliable, high-fidelity antibodies are often lacking in PTM research, these approaches provide unparalleled specificity, drastically reducing false-positive identifications and improper interpretation of results. As a result, this innovative approach has been expanded significantly, with ‘clickable’ metabolic analogs now available to study nearly every class of PTM [100–102].

The metabolic labeling of precursor molecules is the desired approach for the use of chemical biology probes, as they rely on the cells natural mechanism to deposit and regulate PTMs. This approach, however, is not possible for all PTMs as many are sterically restricted or too small (e.g. acetylation, oxidation) to allow for the incorporation of a chemical tag. Reactivity-based protein profiling has been instrumental in the study of numerous PTMs through an agnostic ‘addition by subtraction’ approach [103,104]. Using probes designed to react with unmodified amino acids (e.g. alkynyl-iodoacetamide and Cys), one can assert that a loss of signal stems from PTM occupancy [42,103]. This approach has been utilized extensively and probes have been developed to interrogate the Cys [103], Lys [104], Arg [105], Tyr [106], and Met [107] proteomes. The relative instability of some PTMs also makes sample processing a significant challenge that cannot be addressed with traditional immunoaffinity approaches. S-sulfinylation is a transient oxidation product of a Cys thiol that plays a major role in the redox state of the protein [108]. To overcome this challenge, dimedone-based reagents can be employed to selectively alkylate Cys S-sulfinylation, resulting in stabilization and chemical tagging of this transient PTM [109]. The dimedone warhead can be linked to an alkyne moiety whereby click chemistry can be employed to selectively enrich for S-sulfinylated proteins [109]. Using this approach, 193 protein targets of these labile PTMs have been identified, revealing Cys oxidation as a critical determinant in a host of biological processes, ranging from ER quality control to DNA repair [109].

Perhaps the original application of chemical biology for the study PTMs was the use of metal oxide affinity chromatography (i.e. IMAC) to selectively isolate phosphorylated proteins [110,111]. Here, proteins/peptides are loaded onto a metal oxide column (e.g. TiO_2_), selectively forming a complex with phosphorylated substrates. Modified samples are then eluted and can be monitored using either immunoblotting or LC–MS/MS [112]. This significantly reduces proteome complexity, removing unmodified species and selectively isolating phosphopeptides/proteins, resulting in increased phosphoproteomic depth and identification of lower stoichiometric sites. This approach has also been adapted for the study of phopshoglycerylLys modifications, which possess the necessary negatively charged phosphate moiety for IMAC, improving detection and quantitation of these low abundance PTMs species, which otherwise may have gone undiscovered [19].

## What dictates specificity?

Traditionally, PTMs are viewed as tightly regulated and precisely placed through enzyme-controlled processes. PTM ‘writers’ often recognize a consensus sequence (i.e. a sequence motif) that marks a site for modification. For example, N-linked glycosylation occurs on Asn that reside in the Asn-X-Ser/Thr motif, where X is any amino acid except Pro [113]. This motif is recognized by an oligosaccharyltransferase (the ‘writer’), that transfers a precursor glycan to the target protein. While there are a few instances where N-linked glycosylation may occur outside of this motif (Asn-X-Cys), these PTMs are largely constrained to these sites [114]. Contrary to N-linked glycosylation, O-linked glycosylation occurs on Ser/Thr residues and lacks a consensus sequence, with the parameters that dictate site specificity still under debate [115]. This highlights perhaps the most important questions in the field of PTM research: *What are the parameters that determine site-specificity? How are PTMs that lack a sequence motif regulated?*

**Figure 3.**
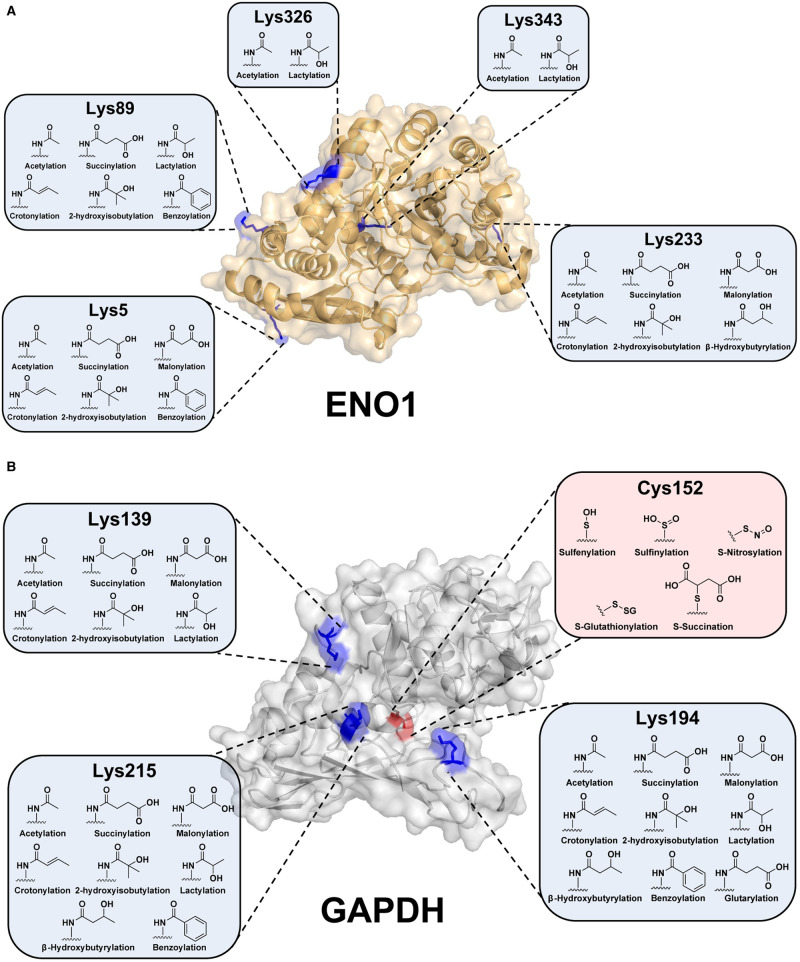
Lys acylations are structurally diverse yet display significant redundancy in modification sites. Proteomic inventories were mined to reveal a striking overlap in modification sites. While many of these sites are surface-exposed, some display a high propensity to modification despite being stearically hindered (e.g. Lys343 on ENO1 and Cys152 on GAPDH). PTM sites were curated from [44,92,116–120,141–147]. ENO1 (PDB: 2SPN) and GAPDH (PDB:3GPD).

An argument could be made that the vast majority of PTMs are derived from non-enzymatic reactions (Table 1) and driven through site availability, local electrostatics, and mass action [18]. In support of this, proteomic inventories often display significant overlap in site-specificity between acylating species, despite lacking a clear sequence motif [72,116]. For example, Lys89 on enolase 1 (ENO1) is a surface exposed residue that is susceptible to acetylation [117], succinylation [116], lactylation [44], crotonylation [118], 2-hydroxyisobutyrylation [119], and benzoylation [120] (Figure 3). While the impact of modification at Lys89 has not been fully elucidated, other peripheral sites have shown significant alterations in stability depending on PTM status. Lactylation of Lys343 results in a significant reduction in stability, while Lys326 displays little to no impact [44]. Although a sequence motif does not exist for most Lys acylations, the propensity for a given site to undergo modification may be explained, in part, by the presence of Cys residues [34]. These Cys readily undergo transient modification (i.e. S-acylation), which is subsequently transferred through an S-to-N acyl transfer onto a nearby Lys residue [16,18,34]. From a biological perspective, these Cys–Lys pairs appear to be detrimental to health as long-lived species (e.g. primates) show less conservation compared with species with shorter lifespans (e.g. mice) [34]. Additional factors may be in play, but this reduced propensity to undergo non-enzymatic Lys acylation may play a larger role in metabolic flux and protein turnover than previously thought, dictating species longevity and/or aging-related disease.

This ‘mass action’ mode of PTM generation is perhaps best explained through the oxidation of Cys residues. Cys152 of glyceraldehyde 3-phosphate dehydrogenase (GAPDH) is heavily prone to modification, susceptible to sulfenylation [121], sulfinylation [122], nitrosylation [123], glutathionylation [124], and succination [125] (Figure 3). Cys152 resides in the active site of GAPDH and is critical for enzymatic activity [42]. This is notable as the PTMs that occupy this site are chemically diverse and are derived from vastly different metabolic sources, yet all serve to reduce GAPDH activity and glycolytic flux [42]. As Cys152 regulates metabolic flux, these oxidations are typically dynamically regulated, many of which display half-lives on the order of minutes [42]. Presumably, Cys152 remains easily accessible as a feedback mechanism to rapidly reduce glycolytic flux and the production of reactive intermediates to prevent further proteome oxidation and cellular damage.

Just as the site-specific addition of a PTM is critical to enzyme activity, efficient removal is also important to restore enzyme function. This action is performed by molecular ‘erasers’ which have been identified for many PTMs (Table 1). When eraser activity is compromised, PTMs accumulate and alter the metabolic landscape. For example, mice lacking the Lys deacylase, SIRT3, display increased Lys acetylation on long-chain specific acyl-CoA dehydrogenase (LCAD), leading to reduced hepatic fatty acid oxidation [126]. The important role of erasers is likely highlighted best when considering PTMs derived from non-enzymatic sources. Lys lactoylation, in addition to mitochondrial acylation, is predominately derived via non-enzymatic means [16,18]. Given the lack of an identified ‘writer’, these PTMs would seemingly modify any available Lys residue through simple mass action. Although this may be the case, there is often significant redundancy in site-specificity between acyl species (Figure 3) [72]. This implies that while the generation of a given PTM may occur stochastically, the sustained occupancy of any given site is regulated through its enzymatic removal. Thus, it is important to consider that while a sequence motif does not exist for all PTMs, mechanisms are likely in place to dictate site-specificity and sustain PTM occupancy as needed.

## Separating regulatory PTMs from bystanders

**Figure 4.**
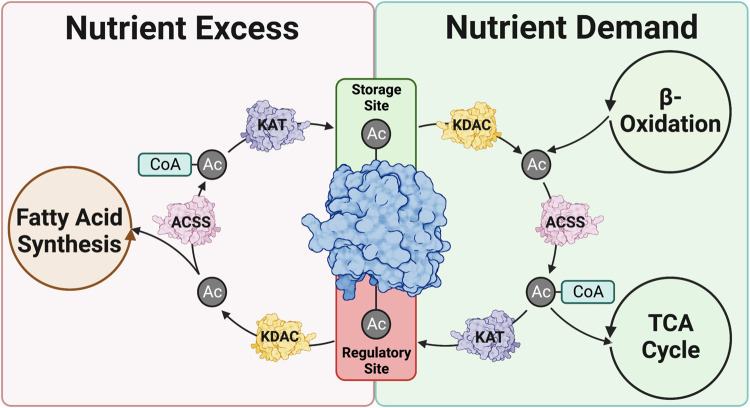
Hypothesized role for non-regulatory acylation sites on proteins. Recent work has demonstrated the presence of non-regulatory acylation sites that can be taken back into acyl-CoA pools and redistributed to regulatory sites. We opine that this may serve as an additional metabolic feedback mechanism during periods of nutrient excess or demand. KDAC, lysine deacylase; ACSS, acyl-CoA synthetase short chain family member; KAT, Lys acytransferase. Created with BioRender.com.

PTMs have historically been viewed as regulatory additions to proteins, providing a rapid response to regulate function [99,117]. In recent years, however, there is mounting evidence that many PTMs may simply be along for the ride, having no measurable impact of protein function [23]. Lys acetylation, for example, is a widely ubiquitous PTM that regulates a host of biological processes, ranging from metabolic flux to chromatin accessibility [6]. Current estimations indicate that 20–50% of all proteins are acetylated, yet many of these modifications do not impact protein function nor physiological outcome [127–129]. This has been elegantly demonstrated on histones, where certain Lys acetylation sites are now proposed to serve as a local store of acetate, readily available for rapid transfer to regulatory sites to alter the epigenomic landscape of the cell (Figure 4) [23]. This repurposing of acetate is an interesting hypothesis that likely holds true outside of the nucleus. KDACs and ACSS isoforms are expressed throughout the cell, making it possible that these reactions take place elsewhere, providing a rapid response to acetylate proteins when acetyl-CoA concentrations are limited. Indeed, as hig hlighted above, protein acylation levels are highly reflective of local acyl-CoA concentrations, so this may serve as an additional mechanism to provide acetyl groups on an ‘as needed' basis [32,33]. Furthermore, the focus on Lys acetylation may be too narrow in scope as KDACs and ACSSs are known to transfer other acyl substrates including propionate [130], crotonate [131], and butyrate [132]. Perhaps the most provocative hypothesis is that these PTMs are placed on proteins during periods of nutrient excess and removed when nutrients are limited, feeding cell metabolism, and maintaining homeostasis (Figure 4).

Multiple factors dictate whether a PTM serves a functional and/or regulatory role. PTMs residing within the active site are often regulatory, altering substrate and/or cofactor binding. This is typically confirmed using recombinant protein, providing kinetic parameters of enzyme inhibition. Recombinant approaches, however, do not provide information on the role of PTMs in cellular trafficking, protein–protein binding, and/or half-life. These questions can be addressed using site-directed mutagenesis, altering the PTM site to either a ‘null’ amino acid, incapable of modification while maintaining necessary electrostatics (e.g. Lys to Arg) or to a PTM mimetic (e.g. Lys to Gln for acetylation) [133,134]. Although his can be informative, site-directed mutagenesis does not provide a direct measure of PTM function, but rather an absence of PTM occupancy. This shortcoming has been addressed through recent advances in genetic code expansion, which provides unparalleled investigations into PTM function on a site-by-site basis [135–137]. Here, unnatural amino acids are precisely incorporated into a protein of interest through a bioorthogonal aminoacyl-tRNA synthetase, which are evolved to recognize and incorporate an amino acid of interest into a target protein [136,137]. This approach has been used to identify lactoylation of Lys343, and not Lys326, as critical modifications mediating ENO1 stability [44]. Although a substantial library of aminoacyl-tRNA synthetases has been generated, this application has not been applied to all PTMs. Furthermore, the modified amino acid monomer must be synthesized and provided to cell culture media, which can prove challenging with more complex PTMs.

An alternative approach for the site-specific incorporation of PTMs into proteins relies on ultrafast split inteins [138,139]. Inteins are a family of protein domains that undergo self-excision as a post-translational modification. By splitting the intein into N- and C-terminal domains (Int^N^ and Int^C^, respectively) it is possible to precisely incorporate a modification of interest into a target protein [139]. To accomplish this, a target protein is tagged with either Int^N^ or Int^C^. Cells are then loaded with a synthetic polypeptide containing the opposing Int. When partner Ints interact, they undergo a rapid association and excision, resulting in chemical ligation of the peptide to the target protein [140]. A major limitation to this approach is the restriction to the N- or C-terminus of the target protein as polypeptides must be synthesized and remain cell-permeable [140]. While issues remain with both codon expansion and intein splicing, they have undoubtedly moved the field forward and provided critical information on the role of PTMs in cell metabolism and chromatin function.

## Conclusions and outlook

PTMs are an integral part of biology, functioning as important signaling molecules to maintain homeostasis and adjust to metabolic and xenobiotic stressors. Consistent with this, there has long been a notion that most PTM sites are regulatory. Although many PTMs do serve this purpose, increasing evidence indicates that many PTMs may simply be along for the ride, providing no alterations in enzyme function. This idea has largely come to light with significant advances in chemical biology approaches. Notably, codon expansion allows for the precise incorporation of a PTM at a single site of investigation, rather than traditional ‘addition by subtraction’ approaches such as site-directed mutagenesis. Collectively, this has led to an intriguing hypothesis that PTMs may serve as a carbon sink, providing metabolic intermediates on demand through their enzymatic removal from protein substrates. As this field continues to experience a rapid expansion, it will be important for studies to maintain high analytical rigor, confirming observations with sound mechanistic studies. Advances in MS approaches have provided much of this added rigor, leading to increased reproducibility sound mechanistic insights into PTM biology.

## Competing Interests

The authors declare that there are no competing interests associated with the manuscript.

## Open Access

Open access for this article was enabled by the participation of University of Arizona in an all-inclusive *Read & Publish* agreement with Portland Press and the Biochemical Society under a transformative agreement with Individual.

## Abbreviations

ACSS: acyl-CoA synthetase short chain family member
ENO1: enolase 1
GAPDH: glyceraldehyde 3-phosphate dehydrogenase
LC: liquid-chromatography
MRM: multiple reaction monitoring
MS: mass spectrometry
PTMs: post-translational modifications
QuARKMod: Quantitative Analysis of Arg (R) and Lys (K) Modifications
SIRTs: sirtuins
